# Trans. of the Provincial Medical and Surgical Association

**Published:** 1842-10

**Authors:** 


### Art. VI.

*Transactions of the Provincial Medical and Surgical Association.* Vol. X.—*London*, 1842. 8vo, pp. 354.

The annual volume published by this admirable society contains its usual variety, though scarcely its usual amount of information. We have first Dr. Streeten's most excellent *Retrospective Address,* and then the conclusion of Dr. Shapter's account of the *Medical Topography of Exeter.* The former of these does not admit of analysis, being itself a condensed analysis; the second we shall have another opportunity of noticing.

The two following papers—that entitled "Observations on the climate of Hertfordshire, compared with that of the neighbourhood of London," by Lieut. Col. Philip Yorke; and the very excellent "Medical Topography of Shrewsbury," by T. Ogier Ward, M.D.—must be passed over for the present.

*Case of a pin passing from the appendix vermiformis into the bladder.* By Dr. Kingdon. This is a curious case. The passage of the foreign body left a fistulous communication between the viscera, through which several worms passed, and escaped by the urethra, occasioning the little patient much suffering. A drawing of the parts is given.

*Case of a tumour developed in the cauda equina.* By Dr. Fisher. The disease originated from an injury of the lower part of the loins. Violent and increasing pains were felt in that region, and at last the patient became paraplegic. On examination after death, a diseased mass was found, entirely surrounded by the divisions of the cauda equina traversed by a few of its nerves. The morbid growth appeared to be developed in the pia mater; the lower portion had the form of a tubercle, presented traces of vascularity in the centre, and had a scirrhous appearance. The other parts were softer, and were composed of a gray, semi-transparent, jelly-like substance, infiltrated amidst cellular tissue, and marked with sanguineous striæ, several of which appeared like true vessels. The original preparation is preserved in the splendid museum of Cambridge; a collection which is second to none out of London, and does infinite credit to the talents, and industry, and public spirit of the Professor of Anatomy and Physiology, Dr. Clark. The parts are here represented in a plate.

*Further observations on the variolæ vaccinæ; with coloured engravings.* By Mr. Ceely. It would not be easy to overrate the importance of these investigations, conducted as they have been with a philosophical discrimination and care that are worthy of all imitation; we shall, therefore, without preface, endeavour to present our readers with as full an abstract as we are capable of laying before them.

*Cases of variolæ vaccinæ occurring in cows and milkers.* Having received information (in October, 1840,) that the tenant of a farm in the village of Oakley, situated at the extreme and n.w. end of the Vale of Aylesbury, had two ruptured vaccine vesicles, caught whilst milking his own cows, some of which he knew were affected with the same disease, Mr. Ceely hastened to the spot in order to enquire minutely into the circumstances. The ruptured vesicles on the hand of the patient (Mr. Pollard, æt. 56, who had never had smallpox or vaccine,) were apparently between the second and third week of the disease. The cows were ten in number, eight milch cows and two sturks. On two of the milch cows there were vestiges on each of not fewer than twenty-five to thirty vaccine vesicles on the teats, and the remains of one on each udder. Two others presented about half that number; and on the fifth there was evidence of only one vesicle on the under part of the teat, which being out of the way of the milker was completely desiccated and entire, forming a characteristic, blackish-brown, oval crust. This crust and two on the udders of the other cows just mentioned were the only perfect ones observed. On the teats all the imperfect crusts had been removed by the manipulations of the milkers, and their places were occupied by florid ulcerations, many of which were manifestly depressed in the centre, and *all* surrounded by the more or less circumscribed, indurated, and elevated integumental boundary which marks the vaccine disease. On the udder of one of the affected milch cows was observed an abundance of the sub-epidermic vesicles or bullæ, which not unfrequently arise during the acme and after the decline of the vaccine disease. The remaining three milch cows had perfectly escaped, and so had one of the sturks; but the other, which proved to be in calf, had several dark-brown crusts on the teats. Another milker was also affected, as will be noticed presently.

Mr. Pollard, the proprietor of the cows, spontaneously stated it to be his opinion *that the animals had been infected from human smallpox effluvia,* and the investigations instituted into this very important point brought to light the following circumstances. It was known that smallpox had been casually introduced into the village where this farm was situated about the commencement of the preceding June, but being promptly met by vaccination, only twelve cases occurred up to the time when the cows exhibited their disease. The last three cases were a woman, aged forty, who had been satisfactorily *inoculated* in infancy by the celebrated Sutton, a young child, and a woman rather beyond the middle period of life. The cottages in which they resided during their illness were situated on each side of, and closely connected with, a long, narrow meadow, comprising scarcely two acres. The first-named patient, though thickly covered with pustules, was not confined to bed after the full development of the eruption; but frequently crossed the meadow to visit the other patients, the woman and child, the former of whom had the disease in its malignant, confluent form, and who died on the 7th September, and was buried the next day.

"On the following day the wearing apparel of the deceased, bed-clothes, bedding, &c. of both patients were exposed for purification on the hedges bounding the close; the chaff of the child's bed was thrown into the ditch; and the flock of the deceased woman's bed was strewed about on the grass within the close, where it was exposed and turned every night and for several hours during the day, till the 13th September—seven days. On that day the above-mentioned eight milch cows and two sturks were turned into this meadow to graze. They entered it every morning for this purpose, and were driven from it every afternoon to be transferred to a distant meadow to be watered and milked, where they remained during the night. Whenever the cows quitted the meadow in question in the afternoon the infected articles above mentioned were again exposed upon the hedges, and the flock of the bed spread out on the grass and repeatedly turned, where it remained until the morning, when the cows were re-admitted. It appears, however, that the removal of the infected articles was not always accomplished so punctually as had been enjoined; for both the proprietors and the milkers affirm that on one occasion, at least, they observed the bed-flock on the grass, and the cows amidst it, and licking it up. The proprietor positively declares, and the milkers corroborate his statement, that the animals were in perfect health on their first entering this close; but within twelve or fourteen days of that event five of the milch cows appeared to have heat and tenderness of the teats, upon which, embedded in the skin, were distinctly felt small, hard pimples, which daily increased in magnitude and tenderness, and in a week or ten days rose into *blisters*, and quickly ran into brown and blackish scabs. At this period, when the teats were thus *blistered* and swollen and very tender, the constitutional symptoms were first observed, viz., sudden 'sinking' or loss of milk, drivelling of saliva from the mouth, and frequent inflation and retraction of the cheeks, staring of the coats, 'tucking up of the limbs,' and 'sticking up' of the back, and rapid loss of flesh. The process of milking was now very difficult, disagreeable, and even dangerous; and on the 14th October, the middle of the third week the detachment of the crusts and loose cuticle, and the abundant discharge of pus on attempting to milk, compelled the milkers to desist for the purpose of washing their hands. Soon after this the cows became by degrees more and more tranquil, as the tenderness and tumefaction of the teats subsided." (p. 214.)

This narrative is particularly interesting. The simultaneous occurrence of the disease in all the subjects (of which the state of the teats bore evidence,) undoubtedly points to the existence of *one* common cause; for it must be borne in mind that, although the vaccine is epizöotic, and attacks one or two cows at *different farms* about the same time, it never, so far as Mr. Ceely's experience extends, has been known to occur simultaneously in so large a number on one farm as in the present instance. Another corroborative circumstance is the fact of the young sturk being affected, because of course the disease could not have been casually communicated to it. But if there were one common cause, where shall we look for it if not in the existence of the variolous effluvia, to which they were all alike exposed, and which certainly would have been quite adequate to the production of smallpox in the human subject? It will be observed also that three of the milch cows escaped altogether, though in every respect situated as the others, and equally liable to have been accidentally infected in the process of milking. This fact, taken in connexion with others of a similar nature, have led Mr. Ceely to the opinion that there exists among cows, as well as among men different degrees of susceptibility to the vaccine.

*Cases of casual vaccine in the milkers.* We shall pass over the first case (Mr. Pollard), because the patient was not seen until the vesicles had burst. The second is more fully narrated. *Case* 2. Joseph Brooks, æt. seventeen, a fine, healthy intelligent young man, who had never been the subject of either variola or vaccine, commenced milking four cows, *one* of which only had the disease, on the 9th October. During ten days he milked six times, the animals having been attended in the intervals by another milker, who had been vaccinated with variola-vaccine lymph, and did not become infected. On the tenth day (October 18th), he felt the cervical glands and lymphatics stiff and tender; and on the 20th found a pimple on the tempora-frontal region, which he could not resist scratching. On the day before that he observed a red pimple on the finger, and next day another, very small, on the thumb. He was not aware of the pre-existence of any injury of the cuticle in those points. On the 21st he had headach, general uneasiness, and pain in the back and limbs, with tenderness and pain along the lymphatics and glands, particularly in the axilla. These symptoms increased until the 23d, when nausea and vomiting took place, after which they gradually declined. The vesicles (of which representations are given,) presented the ordinary appearances, and went through the course which is usual in such circumstances, the centre of each sloughing, and the resulting deep ulcers healing slowly. A small quantity of lymph was obtained from the one on the temple by removing the central crust, and patiently waiting for its slow exudation. It was perfectly limpid, and *very adhesive.* This lymph was used upon the human subject with complete success, both in the primary employment and in the subsequent transfers; the local and constitutional symptoms varying in accordance with the age and temperament of the patient, and the texture and vascularity of the parts. Several of the patients thus vaccinated were tested at the expiration of three months with variola by inoculation, and presented nothing more than a trifling fugitive inflammation at the point of insertion, or a small vesicle, resembling the modified vaccine in form, size, and course, containing a *limpid adhesive* lymph, raised on an indurated base and terminating on the eighth or ninth day in a small, hard, blackish-brown crust, and unattended throughout its course with any constitutional symptoms.

Into Mr. Ceely's remarks upon the early stages of the casual vaccine disease in the cow we need not again enter, having fully noticed them in a previous number, (Brit. and For. Med. Rev., X. 465;) but we should be neglectful of our duty did we fail to impress upon our readers the necessity of a careful attention to the *ulcerative stage,* which has hitherto been less closely observed, but is worthy of much investigation, because it is clear that in many cases our opinion of the existence of the true disease must be chiefly founded upon the phenomena of this period. The difficulties attendant upon this part of the diagnosis are undoubtedly very great, and much time will probably elapse before they are in any considerable degree lessened; but the cause is not hopeless so long as patience and perseverance continue to characterize the members of our profession.

The general points of recognition of the vaccine disease in its other stages being borne in mind, we shall often be able to remark some of their vestiges in the stage of ulceration; but the extent to which this is practicable must depend upon many contingencies. There is nothing so distinctive in the site, figure, or size of the vaccine vesicle as to render the nature of the resulting ulcer (when the crust and all other traces of the original affection have been entirely removed,) at once apparent; more especially when two or three ulcers have been coalescent, or thickly grouped with intervening erysipelatous vesications, in which case the ulcers are generally of large size and irregular shape. True vaccine ulcers are generally distinguishable by a rounded elevation—more or less manifest—of their outer margins, and a circumscribed induration—of greater or less extent—of their base, with a proportionate depression in their centres, sometimes caused by a slough. But these characters are obviously influenced by several circumstances, such as the stage of the disease, the texture of the tissues, and the site of the ulcers; and it should be also remembered that the ulcers resulting from the severer forms of other vesicles may present nearly the same appearances, especially if the tissues are lax and if they have been subjected to much mechanical irritation. If, however, we can be positively assured that the above-mentioned diagnostic conditions have existed in any ulcer for three or four weeks, or longer, especially if it be removed from *severe* casualties, and of a small size, we may fairly presume that it is vaccine. Still it is evident that uncertainty may yet remain, seeing that these are mere questions of degree; but the difficulty is often removed by finding a group of characteristic ulcers on the same animal, or on a series, clearly infected from one another; because such a condition of parts rarely occurs in connexion with the ulcers of other contagious vesicles.

All the cows in this farm were shortly afterwards attacked with the *epizoötic aphtha*, which had long prevailed in the Vale, and which we must briefly notice for the sake of contrast with the description of the vaccine disease given above. This epizoötic seems to be a specific, febrile, eruptive affection, with catarrhal and rheumatic characters, comprising a cold, a hot, and a critical or eruptive stage. Its invasion is generally sudden. The cold stage is very short, and *often unobserved;* the stage of febrile reaction is quickly followed by patches of inflammation on the mucous membrane of the lips, gums, cheeks, tongue, and fauces, and about the nostrils and mouth, with congestion of the conjunctiva. These red patches quickly run into irregular vesications or bullæ, containing a limpid, pale, amber-coloured fluid, and are attended with profuse salivation, difficulty and even temporary inability to masticate, with proportionate constitutional disturbance. Similar vesications are often seen on the teats, especially on the involutions of the skin at their apices, and not unfrequently mammitis results from inflammation of the milk-tubes. Still more frequently they are seen on the skin, where it joins the claws as well as the interdigital membrane, whence they often extend under the horn, creating much mischief, and causing excruciating pain by its detachment, &c. The constitutional symptoms continue for four or five days, when the topical irritation abates and mastication is restored. It attacks all ages, and is often suddenly fatal to the very young, excessive pulmonary and cardiac congestion being found after death. In the milch cow there is a notable diminution of milk ; and in this respect there is a marked difference in the effects of the disease we are now considering and of the vaccine. The quantity of butter lost in the first week of the prevalence of the *aphtha* was thirteen pounds, and in the second week seven pounds; while during the first week of their illness from the vaccine the loss was only six pounds, during the second two, and during the third none.

In the month of June of the next year, Mr. Ceely had the opportunity of investigating a number of cases of *variolæ vaccinæ* at a large dairy farm in the village of Dorton. The disease had existed there for at least three months, and out of forty-eight cows only three had escaped. We shall not enter into any details, excepting so far as to notice the varieties of cicatrices which were observed, and which are of importance in reference to the above-given descriptions of the ulcerative stage. Of the *perfect* cicatrices, some few were irregular, puckered, and uneven; but the majority were regular and well-defined, being oval or circular, and of various sizes, some scarcely perceptible, others as large as a chesnut; their outer margins were slightly elevated and gently rounded off, and their bases a little indurated. On a coloured skin the *whole* of these parts were obviously without pigment. Of the *imperfect* cicatrices, some were irregular and puckered, but most had small, florid, granular, and depressed centres. Some had small, bloody, black, thin, and flat crusts in their centres; and others, again, had in the same place small, brown or blackish brown, thin, secondary, or tertiary crusts. In most of these cases the elevation of the rounded margin and induration of the base was more marked, especially when they were large and irregular.

Two milkers, who had been formerly vaccinated, received the disease in a mild and modified form ; and a young man, who had never had either variola or vaccine, was also infected. His case is detailed at great length, and representations of the vesicles in their different stages are given. We have not space for the details, nor for the results of vaccination with the lymph derived from this subject; but we must beg attention to the following experiment, which appears to us peculiarly interesting:

"Vaccination with the lymph taken from the milker on the 4th of June was performed on two boys, brothers, aged respectively eleven and six, both very fair, with light hair, blue eyes, *red tarsi*, and consequently thin, irritable skins. *On each subject* two vesicles were raised with this '*Dorton*' lymph, two with the '*Oakley*' lymph, and two with the '*variola-vaccine*' lymph. They all advanced in both subjects, *pari passû*, and could not be distinguished either before or after the eighth day. In the younger boy slight headach arose on the *seventh day*. Both complained of local and general symptoms when the areolæ were extending on the *ninth day*. There was nothing remarkable in the size of the vesicles on the tenth day, when the disease was at its acme, and the areolæ were extensively diffused. Eleventh day: areolæ below the elbows. Twelfth day: the elder boy had a very bad night, arm much swollen and inflamed ; the younger had a good night, though his arm was as bad as his brother's. After this period the general symptoms abated; but all the turgid vesicles burst the thin cuticle, the corium sloughed, ulcerated, and threw up loose, spongy granulations not easily repressed." (p. 248.)

The *variola-vaccine lymph* has been so extensively employed, and with such complete success, that little doubt, we should apprehend, can remain upon any mind as to its identity with the genuine vaccine; but for the benefit of those who may yet have some lingering apprehensions we think it right to mention that it has been submitted by Mr. Ceely to the test of variolous inoculation on twenty-two subjects, and that the results were precisely similar to those described by Drs. Jenner and Willan as following the same test after vaccination with the original virus, viz. the production of vesicles closely resembling those of modified vaccine at the point of insertion, occasionally some constitutional irritation, and in one instance a mixed, papular, and papulo-vesicular eruption, analogous to the vesicular lichen sometimes observed after vaccination.

We shall conclude this article with a caution that no lymph should be employed which is not *quite limpid and adhesive*,* and a word of comfort for those who are fearful of employing that which has been recently derived from the cow, on account of their dread of the violent local irritation, but who will perhaps be set at rest when informed that Mr. Ceely has seen "as much local inconvenience in some subjects from the *oldest* as from the *newest* lymph."

The perusal of the present paper only confirms our former estimate of the singular merits of its author. He seems to us to possess the highest qualifications of a philosophical interpreter of nature; and had the Provincial Association no other proofs to adduce of its usefulness—and it has hundreds—the investigations of Mr. Ceely alone would be sufficient warrant for all the labour and all the outlay it has occasioned to its members.

The last paper in this volume is a *Report of Cases at the Chester General Infirmary*: by T. B. Peacock, Esq., which is far too condensed already to suffer any further compression at our hands.

---

### Art. VII.

* M. Dubois of Amiens has stated that efficient concrete vaccine lymph may be recognized by its presenting a beautiful reticulated appearance under the microscope. This appearance, however, would not seem to be diagnostic, for Mr. Ceely has observed it in the *concrete* lymph of the *verrucous vesicle* of the cow, a quite different affection.

